# Management and associated outcomes of COVID-19 infection among Ghanaian autoimmune rheumatic disease patients

**DOI:** 10.4314/gmj.v58i3.2

**Published:** 2024-09

**Authors:** Dzifa Dey, Bright Katso, Derrick Nyame, Saudatu Issaka, Partrick Adjei

**Affiliations:** 1 Department of Medicine and Therapeutics, University of Ghana Medical School, Legon, Accra, Ghana; 2 Rheumatology Unit, Department of Medicine and Therapeutics, Korle-Bu Teaching Hospital, Accra, Ghana

**Keywords:** COVID-19, Autoimmune Rheumatic Disease (AIRD), Management, Self-isolation, Outcome

## Abstract

**Objective:**

This study assessed the prevalence of infection, management strategies and associated disease outcomes of COVID-19 among Autoimmune Rheumatic Disease (AIRD) patients in a teaching hospital in Ghana.

**Design:**

This was a retrospective cross-sectional study.

**Setting:**

Rheumatology Unit, Korle Bu Teaching Hospital.

**Participants:**

Autoimmune Rheumatic Disease patients.

**Results:**

Thirty-one (31) out of approximately 1700 AIRD patients in the unit tested positive for COVID-19, registering a COVID-19 prevalence of 1.82%. The majority, 25(80.6%), were females with a mean ± SD age of 41.7 ± 12.8 years. Systemic lupus erythematosus was the most affected autoimmune rheumatic condition, reporting fever as the commonest COVID-19-related symptom. Most participants, 22(71%), were managed by the “self-isolation”/home management” strategy. In comparison, 7(22.5%) were monitored at the hospital, with both strategies having resulted in complete recovery. The remaining 2(6.5%) patients who managed under “intensive care unit” strategy resulted in mortality.

**Conclusion:**

These findings highlight the relatively low frequency of COVID-19 infection among AIRD patients, the encouraging recovery, and the low severe disease rates observed within this cohort. Additionally, the outcome of self-isolation and home management strategies underscore the importance of personalised approaches to COVID-19 management in this population.

**Funding:**

**None**

## Introduction

The COVID-19 pandemic traces its origins to the emergence of a novel coronavirus, SARS-CoV-2, in Wuhan, Hubei Province, China, in late 2019. This virus is believed to have originated in bats and may have been transmitted to humans through an intermediate host, possibly at a seafood market in Wuhan.^1^,^2^ The World Health Organization (WHO) declared the outbreak a Public Health Emergency of International Concern on January 30, 2020, and a pandemic on March 11, 2020.

The first two cases in Ghana were reported on 12 March 2020 as imported cases from Norway and Turkey, and multifaceted strategies were implemented to mitigate the spread of the virus.^3^ The government first responded by restricting all air travel to Ghana and suspending all public gatherings by March 15, 2020.^4^ Additionally, a ban on social gatherings, which restricted activities and closure of schools, mosques, churches and recreational facilities, was implemented by March 16. Furthermore, the government implemented a mandatory 14-day quarantine for all travellers entering the country from March 17, and by March 22, all borders were closed. This was followed by a partial lockdown of Accra and Kumasi on March 30, and by April 27, it became mandatory for face masks to be worn in all businesses and organisations.^4^,^5^ Despite these efforts, Ghana experienced four waves of the pandemic between 2020 and 2021, reaching peak levels in July 2020 and again in January, August, and December 2021, respectively. Accra in the Greater Accra Region and Kumasi in the Ashanti Region were identified as the two main hotspots during the COVID-19 outbreak in Ghana.^3^ Vaccinations were rolled out in March 2021 in Ghana.^4^

Ghana emerged as one of the motivating stories in Africa regarding scientific achievements and the successful implementation of health measures to ease the pandemic's spread. Remarkably, Ghana conducted the highest number of COVID-19 tests in Africa, second only to South Africa, and holds the top position in the continent for the number of COVID-19 tests conducted per million people. Additionally, scientists from Ghana were among the first in Africa to successfully decode the genomes of the COVID-19-causing virus, SARS-CoV-2.^6^

By January 15, 2022, Ghana had confirmed 153,514 COVID-19 cases, resulting in 1,343 deaths and 9,020 active cases.^4^ The pandemic posed unprecedented challenges for healthcare professionals globally, compelling them to navigate uncharted territory and devise innovative clinical interventions to address the situation, particularly among individuals with underlying health conditions like hypertension, asthma and autoimmune rheumatic disease (AIRD). For instance, Druyan et al. observed that physicians often drew upon theoretical ideas and limited prior pandemic experience to guide their approach.^7^

The swift enforcement of lockdowns and other COVID-19 measures raised significant concerns about the unintended consequences of managing non-communicable diseases.^8^ Moreover, concerns grew regarding the potential adverse outcomes in individuals with AIRD affected by COVID-19.^9^-^11^ The compromised immune state due to the AIRD and immunosuppressive therapies raised questions about infection rates, COVID-19 disease severity and vaccine efficacy.^12^-^15^ Healthcare providers had to carefully balance the risks and benefits of using AIRD medications, leading to varied recommendations regarding managing and treating AIRD patients who contracted the virus.^16^

In Africa, including Ghana, the COVID-19 pandemic also had significant implications for managing AIRDs. A survey of health professionals across Africa reported that many individuals with AIRD experienced disruptions in their access to healthcare services and medications due to lockdowns, reduced clinic hours, and supply chain disruptions.^17^ The international shortage of hydroxychloroquine, a key drug used in the management of AIRDs, was a difficulty experienced by these patients in Africa. This was due to the suggestion that this medication was a possible treatment for COVID-19.^18^,^19^

The 2020 Ghana Standard Treatment Guidelines for the novel coronavirus infection recommended using chloroquine phosphate and hydroxychloroquine for the treatment of all confirmed instances of COVID-19, including symptomatic and asymptomatic, which contributed to its shortage and price increases affecting the usual care of those who needed it for other conditions.^20^

As the pandemic progressed, debates ensued regarding the risk of infection, management strategies to employ, how to navigate medication use in the view of active disease, the risk associated with further immunosuppression, and possible outcomes among AIRD patients.^21^-^24^ These gaps in knowledge underscore the need for comprehensive research to inform future pandemic responses and vaccination strategies.

Some information has been gathered since the pandemic progressed, but the intricate factors influencing the outcomes of individuals with rheumatic diseases still need to be explored.^25^ This is particularly true for Sub-Saharan Africa, where minimal data is available regarding the impact of COVID-19 on AIRD and the potential management modalities that could have influenced outcomes and prepared us for the next pandemic and response to vaccinations. To address this critical knowledge gap, our study investigates COVID-19 infections, management approaches, and associated outcomes among individuals with AIRDs at a teaching Hospital in Ghana during the pandemic.

## Methods

### Study design, setting, and participants

This study design was a retrospective cross-sectional study of autoimmune rheumatic disease (AIRD) patients of the Rheumatology Unit of the Department of Medicine, Korle Bu Teaching Hospital (KBTH) with a confirmed diagnosis of COVID-19 between 1st August 2020 and 31st July 2021. The Rheumatology Unit renders outpatient and inpatient services to patients with various autoimmune rheumatic disorders. Patients come from diverse regions across Ghana and neighbouring West African countries to seek specialist care. The unit presently oversees a cohort of about 1,700 patients with autoimmune diseases, the majority of whom were still actively engaged with the clinic during the pandemic and while this study was ongoing via a recently instituted telemedicine service and in-person clinic reviews.

Patients meeting the American College of Rheumatology and or European League of Associations of Rheumatology EULAR Criteria for the diagnosis of Rheumatoid arthritis, Systemic lupus erythematosus and Mixed connective tissue disease^26^,^27^ who were over 18 years or older were included after providing consent. Various methods were employed to explain the study to them, considering the dynamic circumstances of the early stages of the pandemic.

Participants were identified from the KBTH COVID-19 treatment centre, specifically from the Fevers Unit and Clinical Decision and Treatment Unit (CDTU). Utilising the COVID-19 database of the treatment centre, a list of autoimmune disease patients who tested positive for COVID-19 were accessed, contacted and recruited. Participants were also identified and recruited following responses to continuous electronic surveys sent to the patients managed via telemedicine service during the pandemic. These individuals self-reported on their testing and treatment in other centres or at home. Other positive cases of COVID-19 were also identified from patients self-reporting during their routine in-person rheumatology clinic visits.

The expectation was that other centres would notify the clinic for advice if patients with AIRD were admitted with severe infection. The clinic was the main centre for managing these patients, with only two rheumatologists at the time. All cases included in the study had confirmed Polymerase Chain Reaction (PCR) testing for COVID-19 identified from records or tendered in by patients to confirm diagnosis.

### Data collection methods and analyses

A study questionnaire was designed to capture information on socio-demographic features, such as age, sex, workplace, and city of residence. The questionnaire was also designed to obtain data on participants' COVID-19 status, management strategy or approach, and reported outcomes, all as self-reported by the patients. However, participants' clinical features, such as AIRD diagnoses, disease duration, comorbid conditions, and current medications, were extracted from their medical records using data sheets. Management strategies were thus defined as; “self-isolation/home management,” “monitoring at hospital,” and “intensive care unit”. Outcomes of COVID-19 disease were defined as “complete recovery,” “recovered with post-infection,” and “mortality.”

The questionnaire was designed using Google Forms. Survey links to the forms were posted on the official WhatsApp platform of the rheumatology clinic patients used for telemedicine. In addition, hard copies of the questionnaire were used to collect data from those patients identified at the COVID-19 treatment centres and those receiving care in person during the pandemic.

Data was entered into Microsoft Excel Version 2013 and imported to IBM SPSS Statistics Version 26 for coding and analyses. Frequencies and percentages were used as descriptive summary statistics to present variables.

### Ethical consideration

Ethical approval was sought from the Ethical and Protocol Review Committee of the College of Health Sciences, University of Ghana, Korle Bu Campus, before the commencement of the study with approval number CHS-Et/M.4 – 4.4 /2020-2021. The opening section of the online study questionnaire included an informed consent section where the participants were informed about the purpose and outcomes of the study, their rights as participants, and how their privacy would be protected. The consent statement reads, “By completing this questionnaire, you consent to participate in this study”. All participants were identified with participant IDs to ensure confidentiality.

## Results

Thirty-one (31) out of approximately 1,700 AIRD patients in the Rheumatology Unit of the KBTH tested positive for COVID-19, confirmed by a Polymerase Chain Reaction (PCR) analysis throughout the study period, as shown in Table 1. This indicates a COVID-19 prevalence of 0.0182 among the AIRD patients in the hospital. The mean ± SD age of these patients was 41.7 ± 12.8 years, with the majority 25 (80.6%) females. Of the 31 that tested positive, 17 (54.8%) were diagnosed with Systemic Lupus Erythematosus (SLE), 10 (32.3%) with Rheumatoid Arthritis (RA), and 4 (12.9%) with Mixed Connective Tissue Disease (MCTD). The mean ± SD disease duration was 90.8 ± 60.1 months. The vast majority, 87.1%, were not employed, with a significant portion, 45.2%, residing in the Greater Accra Region during the study period. Out of the total patients, 26 (83.9%) received hydroxychloroquine and prednisolone, while 7 (22.6%) were prescribed methotrexate. Additionally, 6 (19.4%) were on azathioprine, 4 (12.9%) on cyclophosphamide, 2 (6.5%) on mycophenolate mofetil, 15 (48.4%) on omeprazole and 16 (51.6%) on osteo-care. Prevalent comorbidities include hypertension 10 (32.2%), diabetes 5 (16.1%), and chronic kidney disease 2 (6.4%). The patients' most frequently reported COVID-19 symptoms were fever, headache and cough.

**Table 1:** Patient's demographic and clinical characteristics

Item		Total number (N) = 31(%)
**Age, mean ± SD years**	**41.7 ± 12.8 years**	
**Sex**		
**Male**		6 (19.4)
**Female**		25 (80.6)
**Diagnoses**		
**SLE**		17 (54.8)
**RA**		10 (32.3)
**MCTD**		4 (12.9)
**Disease Duration**	**90.8 ± 60.1 months**	
**Comorbidity**		
**Hypertension**		10 (32.2)
**Diabetes**		5(16.1)
**Renal disease**		2 (6.5)
**Others, e.g., asthma, lung disease**		4 (12.9)
**None**		10 (32.2)
**Region**		
**Greater Accra**		14 (45.2)
**Ashanti**		3 (9.7)
**Volta**		6 (19.4)
**Eastern**		4 (12.9)
**Central**		3 (9.7)
**Oti**		1 (3.2)
**Occupation**		
**Administrative assistant**		1 (3.2)
**Civil servant**		1 (3.2)
**HR professional**		1 (3.2)
**Quality assurance officer**		1 (3.2)
**Not working**		27 (87.1)
**Medication**		
**Prednisolone**	Yes	26 (83.9)
	No	5 (16.1)
**Hydroxychloroquine**	Yes	26 (83.9)
	No	5 (16.1)
**Methotrexate**	Yes	7 (22.6)
	No	24 (77.4)
**Azathioprine**	Yes	6 (19.4)
	No	25 (80.6)
**Cyclophosphamide**	Yes	4 (12.9)
	No	27 (87.1)
**Mycophenolate Mofetil**	Yes	2 (6.5)
	No	29 (93.5)
**Omeprazole**	Yes	15 (48.4)
	No	16 (51.6)
**Calcium**	Yes	16 (51.6)
	No	15 (48.4)
**COVID-19 symptoms**		
**Fever**	Yes	22 (71.0)
	No	9 (29.0)
**Headache**	Yes	22 (71.0)
	No	9 (29.0)
**Cough**	Yes	21 (67.7)
	No	10 (32.3)
**Sore throat**	Yes	12 (38.7)
	No	19 (61.3)
**Muscle pain**	Yes	11 (35.5)
	No	20 (64.5)
**Difficulty breathing**	Yes	9 (29.0)
	No	22 (71.0)
**Chills**	Yes	8 (25.8)
	No	23 (74.2)
**Runny nose**	Yes	7 (22.6)
	No	24 (77.4)
**Loss of smell/taste**	Yes	7 (22.6)
	No	24 (77.4)
**Diarrhea**	Yes	4 (12.9)
	No	27 (87.1)
**Abdominal pain**	Yes	3 (9.7)
	No	28 (90.3)
**Nausea/vomiting**	Yes	3 (9.7)
	No	28 (90.3)

Table 2 shows that the primary COVID-19 management approach was “self-isolation/home management,” accounting for 71% of all participants. Of those managed by this approach, 13 (59.1%) had SLE, 8 (36.4%) had RA, and 1 (4.5%) had MCTD. Monitoring at the hospital as a management approach came second to self-isolation, reported in 7 (22.5%) of the patients, including 3 (42.9%) SLE cases, 1 (14.2%) RA case, and 3 (42.9%) cases of MCTD. The “self-isolation” and “monitoring at hospital” management strategies resulted in complete recovery. Two participants, who accounted for 6.5% of the total (one with SLE and the other with RA), were managed under the “intensive care unit” strategy, resulting in mortality for both. The mortality rate among the positive COVID-19 patients was hence 6.5%.

**Table 2:** COVID-19 management strategies and their outcomes

Management Type	COVID-19 Disease Outcome [Total N = 31]			
	Complete Recovery		Recovered with post-infection	Mortality
	n (%)		n (%)	n (%)
**“self-isolation”**		22 (71.0)		
**SLE**	13 (42.0)		0(0)	(0)
**RA**	8 (25.8)		(0)	(0)
**MCTD**	1 (3.2)		(0)	(0)
**“Monitoring at hospital”**		7 (22.6)		
**SLE**	3 (9.7)		(0)	(0)
**RA**	1 (3.2)		(0)	(0)
**MCTD**	3 (9.7)		(0)	(0)
**“Intensive care unit”**		2(6.4)		
**SLE**	(0)		(0)	1(3.2)
**RA**	(0)		(0)	1 (3.2)
**MCTD**	**(0)**		**(0)**	**0 (0)**

## Discussion

Since the COVID-19 outbreak, due to several established factors, much attention has been paid to safeguarding individuals in high-risk groups, such as those who suffer from systemic autoimmune rheumatic disease and other non-communicable diseases.^28^,^29^ Studies about the SARS-CoV-2 virus infection in AIRD patients have been the subject of small, primarily regional characterisation studies. As a result, the outcomes of COVID-19 in individuals with AIRD are still not well characterised.

This study represents one of the few original African studies examining COVID-19-related impact on patients with AIRDs. Earlier studies exploring the topic in Africa tried to describe institutional challenges such as the shutdown of rheumatological services and reduction in clinical visits due to COVID-19.^17^,^30^ By determining the outcome of COVID-19 in AIRD patients with various management strategies. Our study represents a pivotal contribution to this area, especially for the African population. We focused on patients managed at a single centre and those registered in that centre; however, considering the dynamics of the early stages of the pandemic and the management strategies, not all patients needed to come to the hospital for care. Therefore, the study had a regional outlook, as some patients were managed at home, for example, outside the study centre.

We identified COVID-19 infection in 1.8% of the AIRD patient population at the Korle Bu Teaching Hospital (KBTH), the main referral hospital for patients with AIRD across Ghana. A similar percentage was reported in one of the earliest studies, with the incident cases of COVID-19, including the definite diagnosis and highly suspected cases being 1.5% among the autoimmune systemic disease patients.^31^ These percentages are lower than findings reported in a single-centre study conducted in Israel around the same period, where 3.6% of the patients were identified as COVID-19 positive.^7^ Later studies from much larger cohort studies and meta-analyses in other populations outside Africa showed much higher percentages (7.9%), as was discovered by Felten et al., and about 11%, as found by Akiyama et al.^15^,^32^

In the current study, commonly reported COVID-19 symptoms experienced by the patients were fever, headache, and cough. The symptoms reported here were consistent with those found to be most common in other studies and align with the top potential symptoms of COVID-19 as recognised by both the World Health Organization and the Centers for Disease Control and Prevention.^32^,^33^ The two mortality cases in our study were 64 years old for the RA case and 38 years for the SLE case.

While an earlier study found that older age is a risk factor for severe COVID-19 outcomes in these individuals,^34^ were not explored due to the small sample size. Most of the COVID-19 infections reported in this study were from the Greater Accra region. This finding is consistent with national data on COVID-19 infection from the Ministry of Health, which shows that the Greater Accra region had the bulk of national COVID-19 infection cases reported among the 16 regions of Ghana.

Systemic lupus erythematosus (SLE) patients were the most impacted among the AIRDs in this study, likely due to their higher representation, constituting approximately 55% of the AIRDs cohort at the Rheumatology Unit, KBTH. Notably, earlier research findings suggested that the specific type of autoimmune disease did not correlate with an increased risk of infection or severe COVID-19 outcomes.^22^

Females constituted the majority of COVID-19 cases in the study, consistent with findings from other studies among autoimmune diseases, indicating a higher likelihood of infection among women.^22^,^23^,^32^. However, national epidemiological studies on the COVID-19 outbreak in Ghana have shown a male predominance^3^,^35^,^36^, highlighting the need for further research to elucidate the reasons behind this gender difference within specific patient groups compared to national statistics. Understanding these disparities is crucial for informing disease management strategies and public health interventions.

The “self-isolation”/home management” and “monitoring at hospital” management strategies produced outcomes of complete recovery, indicating their effectiveness in managing COVID-19 among individuals with AIRDs. This discovery agrees with Felten et al., where the authors found that most (79%) of the confirmed COVID-19 cases were home-managed, just like in our study, and no mortality was recorded from their research relating to this management strategy. However, unlike our findings, their research did not report any mortality among patients receiving “intensive care unit” (ICU) management. Rather, Felten et al. noted a fatality associated with conventional care or “hospital monitoring”, which was not the case as reported by our research with this same management strategy.^32^ Though “self-isolation” or home management by default would be the management strategy for less severe cases, better outcomes are expected in such a group.

Commonly reported comorbidities in our study were hypertension and diabetes, and these are frequently encountered in individuals with autoimmune diseases.^37^

The presence of comorbidities, such as hypertension and diabetes, has been implicated in increasing the risk of severe COVID-19 outcomes.^38^ Hypertension not only exacerbates the underlying inflammatory processes in autoimmune diseases but also contributes independently to endothelial dysfunction and vascular damage, which are key features of severe COVID-19 pathology.^39^ One of the two cases in our study with poor outcomes was an older adult diagnosed with hypertension and diabetes along with gout. These features are similar to the clinical descriptions of patients who exhibited the worst COVID-19 outcomes in previous studies.^36^ However, it is important to acknowledge that this study did not explore the independent risk of hypertension and diabetes for poor COVID-19 outcomes among the patients, largely due to the small sample size.

The SLE and RA patients who died were both on hydroxychloroquine and prednisolone. Additionally, the SLE patient was on cyclophosphamide while the RA patient was on methotrexate, both medications used in severe and active diseases. It has been reported that underlying disease activity may increase the risk of COVID-19 complications more than glucocorticoid use. Moreover, medications associated with poor COVID-19 outcomes in previous studies were Sulfasalazine and B cell-depleting therapies such as Rituximab.^40^ Based on the available data, conventional synthetic DMARDs did not increase the risk of poor outcomes in COVID-19. This is in line with the recommendations from ACR and EULAR, which advise continuing the current treatment with these agents if there is no known exposure to SARS-CoV-2 to reduce the risk of flares except for Rituximab.^41^,^42^

This study is essential, but it is important to acknowledge its limitations. Missing information on the therapeutic association of some anti-rheumatic immune-modulating medications, such as hydroxychloroquine 32,43 or glucocorticoids, which may suppress the cytokine storm^44^ associated with COVID-19 severity, is noted.^45^ Patients who defaulted clinic reviews or were lost for follow-up due to the COVID-19 pandemic were excluded. However, they comprise only a small percentage of the total AIRD cohort, so their influence on the outcomes is minimal. Given the patient population, it was difficult to examine the infection rate per the various pandemic waves in the country. Again, this study was conducted just before the introduction of the COVID-19 vaccines and its impact was not assessed. Moreover, participants, especially those who self-responded to surveys, may likely be subject to recall bias concerning information regarding COVID-19 management and their actual AIRD management. However, efforts were made to minimise this bias through clear instructions on the questionnaire.

The number of participants who tested positive was small, limiting the generalizability of the findings, but it also buttresses the fact that AIRD patients, as reported through education, observed isolation rules and were less likely to be infected.^46^

## Conclusion

Overall, the findings of our study highlight the relatively low frequency of COVID-19 infection among AIRD patients, the encouraging recovery, and the low severe disease rates observed within this cohort. Additionally, the positive relationship between self-isolation/home management strategies and complete recovery suggests the importance of personalised approaches to COVID-19 management in this population. These findings underscore the significance of proactive measures in mitigating the impact of COVID-19 within this vulnerable population. Tailored interventions focusing on self-care and isolation could significantly contribute to optimising patient outcomes and reducing the severity of complications in AIRD patients during the pandemic.
